# Insight into diversity of bacteria belonging to the order *Rickettsiales* in 9 arthropods species collected in Serbia

**DOI:** 10.1038/s41598-019-55077-y

**Published:** 2019-12-10

**Authors:** Kun Li, Maja Stanojević, Gorana Stamenković, Bojan Ilić, Milan Paunović, Miao Lu, Branislav Pešić, Ivana Đurić Maslovara, Marina Siljic, Valentina Cirkovic, Yongzhen Zhang

**Affiliations:** 1 Department of Zoonoses, National Institute for Communicable Disease Control and Prevention, Chinese Center for Disease Control and Prevention, Beijing, China; 2 University of Belgrade Faculty of Medicine, Institute of Microbiology and Immunology, Belgrade, Serbia; 3 University of Belgrade, Institute for Biological Research, Siniša Stanković, Department for Genetic Research, Belgrade, Serbia; 4 University of Belgrade, Faculty of Biology, Institute of Zoology, Department of Animal Development, Belgrade, Serbia; 5 Natural History Museum in Belgrade, Belgrade, Serbia; 6 Institute for Biocides and Medical ecology, Belgrade, Serbia; 7 Shanghai Public Health Clinical Center & Institute of Biomedical Sciences, Fudan University, Shanghai, China

**Keywords:** Pathogens, Bacterial genetics

## Abstract

*Rickettsiales* bacteria in arthropods play a significant role in both public health and arthropod ecology. However, the extensive genetic diversity of *Rickettsiales* endosymbionts of arthropods is still to be discovered. In 2016, 515 arthropods belonging to 9 species of four classes (Insecta, Chilopoda, Diplopoda and Arachnida) were collected in Serbia. The presence and genetic diversity of *Rickettsiales* bacteria were evaluated by characterizing the 16S rRNA (*rrs*), citrate synthase *(gltA*) and heat shock protein (*groEL*) genes. The presence of various *Rickettsiales* bacteria was identified in the majority of tested arthropod species. The results revealed co-circulation of five recognized *Rickettsiales* species including *Rickettsia*, *Ehrlichia* and *Wolbachia*, as well as four tentative novel species, including one tentative novel genus named *Neowolbachia*. These results suggest the remarkable genetic diversity of *Rickettsiales* bacteria in certain arthropod species in this region. Furthermore, the high prevalence of spotted fever group *Rickettsia* in *Ixodes ricinus* ticks highlights the potential public health risk of human *Rickettsia* infection.

## Introduction

The order *Rickettsiales*, belonging to the class Alphaproteobacteria, represents a rather diverse group of endosymbiotic gram-negative bacteria. The order *Rickettsiales* comprises of two established families, *Anaplasmataceae*, *Rickettsiaceae*, and 2 tentative families, *Candidatus* Holosporaceae and *Candidatus* Midichloriaceae^1,2^. Within the order, bacteria from the genera *Rickettsia*, *Ehrlichia*, *Anaplasma* and *Candidatus* Neoehrlichia are the most studied due to their known pathogenicity to animals and humans. They are strikingly widespread in various hosts of different kingdoms, including Animalia (arthropods and vertebrates), Protista and even Plantae^2–4^. Nevertheless, along with vertebrates, arthropods are considered to be among the primary hosts for most members of the order *Rickettsiales*. For the genera *Rickettsia*, *Anaplasma, Ehrlichia* and *Candidatus* Neoehrlichia, Ixodid ticks are important natural reservoirs and vectors. Furthermore, *Wolbachia* is one of the most common parasitic microbes and its hosts cover a great many of arthropod species, including a large proportion of insects and some nematodes^5^. Notably, some of these endosymbiotic bacteria are mutualistic with their arthropod hosts. In some cases, the infection by these bacteria is beneficial or even essential for the development and/or reproduction of their hosts (e.g. manipulating sex ratio, effecting parthenogenesis, influencing the fitness, etc)^6^.

Arthropods harbor a substantial diversity of bacterial endosymbionts. In recent years, the improving techniques of pathogen screening have contributed to a remarkable increase in our knowledge of the number and diversity of endosymbiont bacteria from arthropods. Especially, in arthropod species highly relevant to human health, such as ticks and mosquitoes, bacterial endosymbionts have been extensively investigated^7^. However, they have been much less studied in most other arthropod species so far. Hence, the considerable genetic diversity of *Rickettsiales* endosymbionts coupled to the enormous wealth of arthropod species is still to be discovered.

In Serbia, a number of arthropod borne pathogens are known to be present, both endemic and epidemic, with existing reports of both human and reservoir/vector infection, e.g. arboviruses such as Crimean-Congo haemorrhagic fever virus, west-Nile virus, etc^8,9^. Notably, data about bacterial agents associated to arthropods in Serbia, endosymbiotic or other, are still rather scarce^10,11^.

The aim of this study was to screen for the presence of *Rickettsiales* bacteria in several arthropod species (including mosquitoes, ticks, bedbugs, millipedes, centipedes) collected in Serbia.

## Material and Methods

### Collection and processing of arthropods

**Figure 1:**
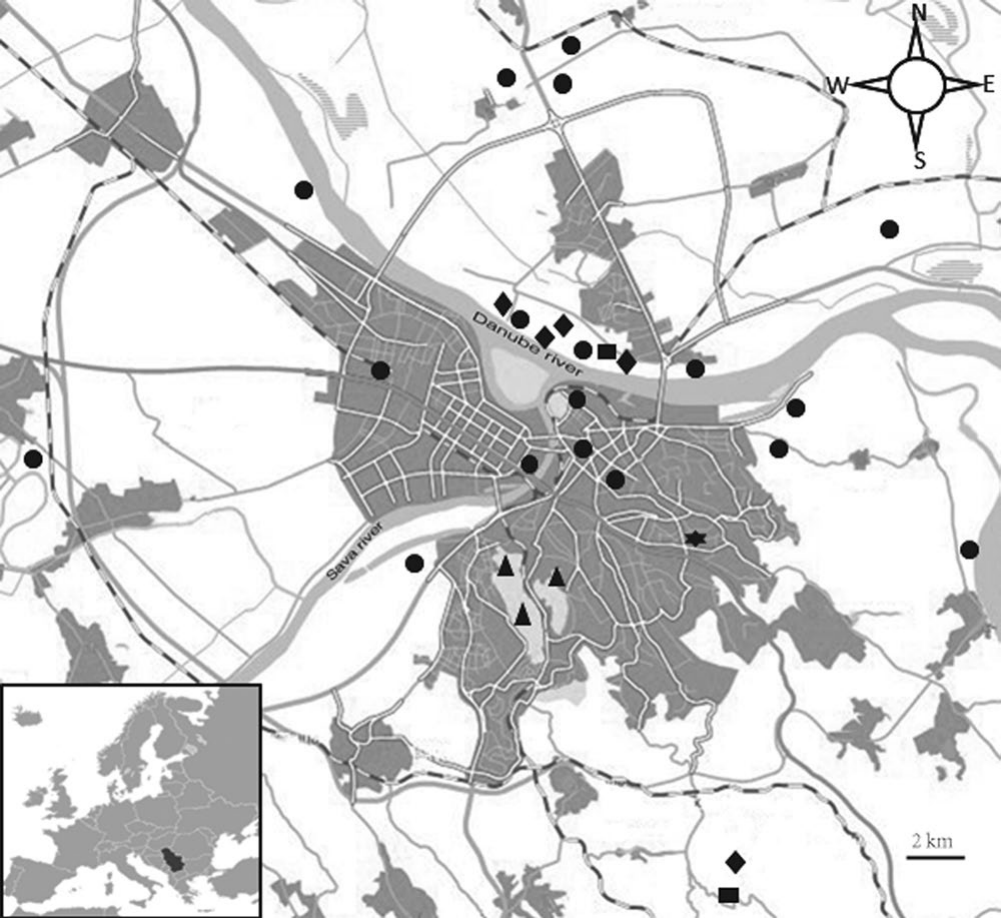
A map of Belgrade city, Serbia, showing the location of sites from which arthropods were collected.

During June to September of 2016, arthropod samples were collected from 27 sites in Serbia. Majority of collection sites (23/27) was within the city of Belgrade and its nearest surroundings (20.40°E, 44.59°N) (Fig. 1). Two sites were in the Pannonian Plain, around 150 km to the north-west and 70 km to the northeast of Belgrade (N44.935 E21.136 and N45.455 E19.220, respectively), whereas two sites were in the southern part of the country (N42.924 E22.169 and N42.710 E22.342, respectively) (Table S1). For mosquitoes, arthropod collection was performed by using BG sentinel trap (A kind of widely used mosquito monitoring trap) with dry ice container. Ticks were collected by flagging over vegetation and grassland. Other arthropod species included in the study were directly picked from the ground. Notably, flea specimens were the only ones collected from infested animals – bats of the species *Nyctalus noctula* that were subsequently released to freedom (in accordance with the protocol approved by the Ethical Committee). Upon collection, the arthropods were immediately stored at −80 °C, while transport to China CDC was performed in nucleic acid preservative (DNA/RNA Shield, Zymo Research, Irvine, CA, USA) at room temperature until RNA/DNA extraction. Upon collection the arthropods were morphologically identified, and then further confirmed by sequencing the 18S ribosomal RNA (18S rRNA) gene.

### Sample processing, DNA extraction, PCR and DNA sequencing

For nucleic acid extraction the arthropods were removed from the DNA/RNA shield and washed three times with phosphate-buffered saline (PBS). Larger specimens, of a size in the range of centimeters and more were cut into pieces. All samples were homogenized in 0.8 ml PBS solution using Retsch MM400 homogenator. The suspension was subjected to 2500 g centrifugation for 5 min, and the supernatant was collected for DNA extraction. QIAamp DNA Mini Kit (Qiagen GmbH, Hilden, Germany) was used for DNA extraction according to the manufacturer’s instructions.

Screening for *Rickettsia*, *Wolbachia*, *Ehrlichia* and *Anaplasma* was performed by nested or semi-nested PCR targeting a conserved sequence of the 16S rRNA (*rrs*) gene (1509 bp), as described previously^12,13^. For amplification of partial heat shock protein (*groEL*) (614 bp) and citrate synthase (*gltA*) (760 bp) genes a nested PCR protocol was applied, with the use of primers that were either previously described^13^ or designed for the current study (Table S2). In positive samples, for the purpose of further exploration of phylogenetic positioning of bacterial strains representing prospective novel genera, partial RNA polymerase β-subunit-encoding (*rpoB*) gene sequence (2673 bp) was also amplified, by DNA walking (Primers shown in Table S2).

PCR amplicons were analyzed by electrophoresis in 1.0% agarose gels. The bands were compared to standard molecular size DNA ladders. Positive PCR products shorter than 800 bp were subjected to direct Sanger sequencing (Sangon Biotechnology Company, Shanghai, China). Purified DNA fragments of a size longer than 800 bp were cloned into cloning vector pMD19-T (TaKaRa, Dalian, China), transformed into *E. coli* and plated onto culture dish. The obtained clones were picked and sent for Sanger DNA sequencing (Sangon Biotechnology Company, Shanghai, China).

### Sequence data and phylogenetic analyses

Nucleotide sequence similarities between the recovered bacterial sequences and those existing in the GenBank were calculated using DNAStar (DNASTAR, Inc., Madison, WI). For phylogenetic analysis, the recovered sequences were aligned with reference sequences using ClustalW (default parameters) within the MEGA program, version 5.2^14^. The best-fit evolutionary model for all sequence alignments was determined using jModel Test^15^. The General Time Reversible (GTR) nucleotide substitution model, with a gamma (Γ)-distribution model of among-site rate variation and a proportion of invariable sites (i.e. GTR + Γ + I), was found to be the best-fit model for these sequences. Phylogenetic trees were then estimated using the Maximum Likelihood (ML) method implemented in PhyML, version 3^16^. To estimate the support for individual nodes, 1000 bootstrap replicates were obtained under the same procedure. All trees were mid-point rooted for clarity only.

## Results

### Arthropod collection and *Rickettsiales* bacteria detection

During the four months study period, a total of 515 arthropods (including ticks, mosquitoes, bedbugs, centipedes, millipedes, fleas) were collected from 27 sites in Serbia (Fig. 1). In total, nine arthropod species were identified among collected specimens, namely *Culex pipiens, Ixodes ricinus, Cimex lectularius, Polydesmus complanatus, Clinopodes flavidus, Cryptops anomalans, Pachyiulus hungaricus, Strigamia sp., Ischnopsyllus* sp. (Table 1). Species identification based on morphological examination and sequence analysis of the 18S rRNA gene was obtained for all collected arthropods. The collected arthropods were divided into 39 pools of different size (ranging from 1–31 specimens per pool) according to species, trapping site location and collection date. The pool size, species, collection date and geographic distribution are described in Table S1.

**Table 1:** Prevalence of *Rickettsiales* bacteria in arthropods collected in Belgrade, Serbia, during 2016.

Species	*Rickettsia monacensis*	*Rickettsia helvetica*	*Rickettsia* spp.	*Rickettsia* endosymbiont of *Polydesmus complanatus*	*Ehrlichia HF*	*Wolbachia pipientis*	*Wolbachia* endosymbiont of *Cimex lectularius*	*Wolbachia* endosymbiont of *Ischnopsyllus* sp.	*Candidatus* Neowolbachia serbia	Total
*Culex pipiens*	0	0	0	0	0	5	0	0	0	5/18
*Ixodes ricinus*	3	1	1	0	1	0	0	0	0	6/8
*Cimex lectularius*	0	0	0	0	0	0	5	0	0	5/5
*Polydesmus complanatus*	0	0	0	1	0	0	0	0	0	1/1
*Clinopodes flavidus*	0	0	0	0	0	0	0	0	0	0/1
*Cryptops anomalans*	0	0	0	0	0	0	0	0	0	0/3
*Strigamia* sp.	0	0	0	0	0	0	0	0	1	1/1
*Pachyiulus hungaricus*	0	0	0	0	0	0	0	0	0	0/1
*Ischnopsyllus* sp.	0	0	0	0	0	0	0	1	0	1/1

Amplicons of the *Rickettsial rrs* gene were successfully generated and DNA sequences obtained from 19/39 arthropod pools, comprising six different arthropod species. Bacterial DNA was detected in 5/18 *C. pipiens* (27.78%), in 6/8 *I. ricinus* (75.00%), in 5/5 *C. lectularius* (100.00%), in 1/1 *Strigamia sp*., in 1/1 *P. complanatus* and in 1/1 *Ischnopsyllus* sp. pools, whereas tested samples of three species (*Clinopodes flavidus, Cryptops anomalans, Pachyiulus hungaricus) were found negative*. A total of 30 new bacterial sequences were recovered and have been deposited in the GenBank, with the accession numbers MH618374 to MH618404. The obtained sequences exhibited similarity in the *rrs* gene with those of the genera *Ehrlichia*, *Rickettsia*, and *Wolbachia*, with percentage identities from 91.2–100%. Among the samples found positive for *gltA* and *groEL* gene sequences, within each species, the similarities between the sequences recovered here and known reference sequences from GenBank varied from 71.6% to 100% for the *gltA* gene sequences, and from 71.3% to 100% for the *groEL* gene sequences.

### Diverse *Ehrlichia* and *Rickettsia* bacteria circulating in arthropods

**Figure 2:**
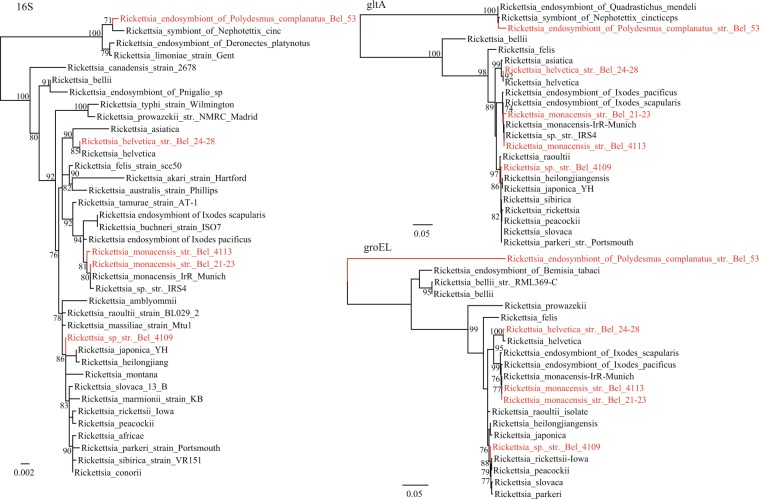
Phylogenetic analysis of *Rickettsia* strains based on nucleotide sequences of *rrs*, *gltA* and *groEL* genes, as well as those retrieved from GenBank.

Based on molecular detection and phylogenetic analysis of the target *rrs, gltA* and *groEL* genes, the presence of one *Ehrlichia* and four *Rickettsia* bacterial species was revealed in the analyzed arthropods from Serbia (Table 1, Fig. 2). On the basis of gene similarity and positioning in the phylogenetic trees, the newly detected *Ehrlichia* sequence in *I. ricinus* ticks was identified as *Ehrlichia* sp. HF, whereas the newly detected *Rickettsia* included three species: *Rickettsia monacensis, Rickettsia helvetica* and one *Rickettsia* sp. that remained unassigned to any defined species cluster. In phylogenetic trees for all three studied genes, *rrs, groEL* and *gltA*, it was found within a larger monophyletic group that, among others, included strains of spotted fever group *Rickettsia* species: *Rickettsia raoultii, Rickettsia japonica, Rickettsia heilongjiangensis, Rickettsia slovaca, Rickettsia parkeri*. However, it clustered separately, indicating a separate, new species. *R. monacensis* was found in 3 pools, while *R. helvetica, Ehrlichia* sp. HF and a *Rickettsia* sp. were identified in one pool each of *I. ricinus* ticks. These findings implied the co-circulation of various bacteria of spotted fever group (SFG) *Rickettsia* in Serbia.

Furthermore, a divergent *Rickettsia* strain was identified from *P. complanatus*. As indicated in the phylogenetic trees, the *rrs* and *gltA* genes of *Rickettsia* endosymbiont sampled from *P. complanatus* clustered with those of *Rickettsia* symbiont of *Nephotettix cincticeps* with the identity of 99.62% and 98.11% respectively. However, the *groEL* of *Rickettsia* endosymbiont of *P. complanatus* formed an independent branch in the phylogenetic tree with highest 80.52% identity to *Rickettsia bellii* and *Rickettsia* endosymbiont of *Bemisia tabaci*, suggesting that it may represent a novel species (*Rickettsia* endosymbiont of *P. complanatus)* belonging to *Rickettsia* Torix group.

### Diverse *Wolbachia* and *Neowolbachia* bacteria circulating in arthropods

**Figure 3:**
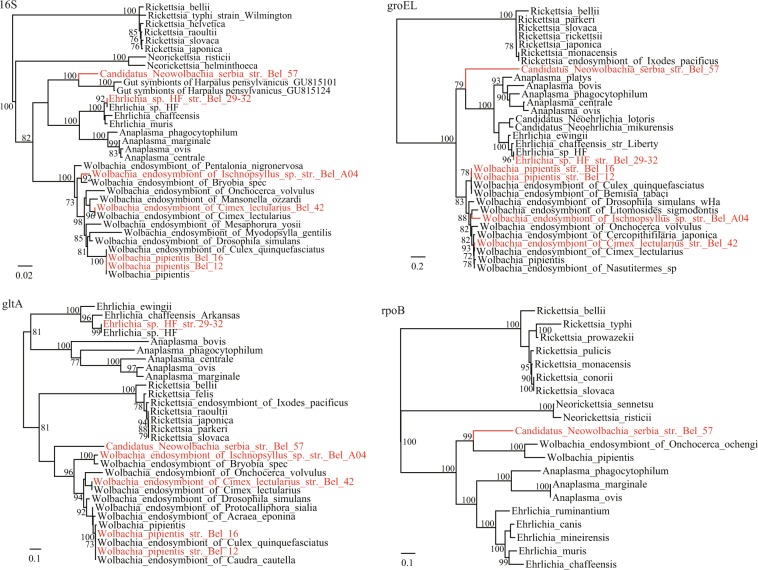
Phylogenetic analysis of *Wolbachia* and *Candidatus* Neowolbachia strains based on nucleotide sequences of *rrs*, *gltA*, *groEL* and *rpoB* genes, as well as those retrieved from GenBank.

*Wolbachia* bacterial DNA was found in arthropod pools of *C. pipiens L* and *C. lectularius*, but also in a pool comprised of specimens of *Ischnopsyllus sp*. (Table 1). In the *rrs*, *gltA* and *groEL* gene trees (Fig. 3), the *Wolbachia* sequences from Serbian arthropods fell into three lineages, *W. pipientis*, *Wolbachia* endosymbiont of *C. lectularius* (*w*Cl), and *Wolbachia* endosymbiont of *Ischnopsyllus* sp. (*w*Is) (Fig. 3). Notably, *Ischnopsyllus* hosts have never been described previously to be infected by *Wolbachia*. In this study, we identified the *w*Is Bel-A04 strain, which was phylogenetically related to the *Wolbachia* endosymbiont of *Bryobia spec* (*w*Bs) (Fig. 3). The *rrs*, *gltA* and *groEL* genes of *w*Is Bel-A04 showed moderate similarity to the known *Wolbachia* sequences, with highest nucleotide similarity of 98.3%, 93.8% and 87.6% in the *rrs*, *gltA* and *groEL* genes, respectively, to the *Wolbachia* endosymbiont of *Bryobia spec* (*w*Bs) and *Wolbachia* endosymbiont of *Nasutitermes sp*. (*w*Ns) (Fig. 3). Although the *Wolbachia* surface protein (*wsp*) gene or other key genes were not obtained, we propose that it may represent a novel *Wolbachia* species (*Wolbachia* endosymbiont of *Ischnopsyllus* sp).

Remarkably, a divergent bacterial strain from *Strigamia sp*. was identified, of which the *rrs* gene showed highest similarity of 91.2% and 91.0% to *Candidatus* Ehrlichia shimanensis and *Wolbachia* endosymbiont of *Mesaphorura yosii*, respectively. As to the *gltA* and *groEL* genes, 71.6% and 71.3% similarity to the known *Wolbachia* strains was observed, respectively. Notably, the *rpoB* gene of this strain shares 74.1% homology to *Wolbachia* and 73.9% homology to *Ehrlichia* (Table S3). In the phylogenetic trees based on the *rrs*, *gltA* and *rpoB* genes, the Bel-57 strain formed a distinct lineage and is most closely related to *Wolbachia* (Fig. 3). Based on the nucleotide identity and phylogenetic analysis, we propose that this *Wolbachia*-like strain might belong to a novel genus mostly related to *Wolbachia*. Therefore, we tentatively nominate it *Candidatus* Neowolbachieae and the strain name *Candidatus* Neowolbachia serbia Bel-57.

## Discussion

The phenomenon of endosymbiosis, or one organism living within another, is considered to have substantially influenced the evolution of numerous species, with continuing impact^4,17^. Arthropods are believed to be one of the main reservoirs of bacterial endosymbionts which have co-speciated and co-evolved with their arthropod hosts for a long time^4,17^, leading to enormous bacterial species diversity.

Here, we present a study exploring the presence of bacteria belonging to the order *Rickettsiales* in 9 species of arthropod samples from Serbia, collected over several months period in different sites, mainly in the capital city of Belgrade. Belgrade is located at the confluence of Sava river to Danube river, where the Balkan Peninsula meets the Pannonian Plain. With a moderate continental climate of four seasons, sufficient rainfall and enough warm weather condition to support diverse flora and abundant animals, such as diverse arthropods and mammals, environment in Belgrade could provide wide host reservoir for various bacterial endosymbionts. So far, Serbia and Belgrade comprise a known endemic region for arthropod borne pathogens such as arboviruses - autochthonous cases of arboviral diseases have continuously been reported^8,9^. Nevertheless, the real epidemiological situation regarding arthropod borne infections might be underestimated, since details about arthropod related bacterial agents in Serbia are largely unknown^18^. Majority of previous studies have explored the presence of bacteria in ticks^19–21^. In the presented study, species of arthropods derived from four different classes (Insecta, Chilopoda, Diplopoda and Arachnida) were collected from several sites in Serbia and found to contain considerable diversity of *Rickettsiales* bacteria. Arthropod species included mosquitoes, ticks, bedbugs, millipedes, centipedes, fleas, whereas among the found bacteria, various *Rickettsia*, *Ehrlichia*, *Wolbachia* and a prospective novel genus designated *Neowolbachia gen. nov*. were identified. Of note, arthropod samples preparation included washing in PBS, but not using 70% ethanol, hence a possibility of environmental contamination might exist. However, in view of the detection rate and scope of the found species, we believe that these endosymbiontic bacterial sequences are from the tested arthropods themselves. Considering that the current survey for endosymbiotic bacteria included only 9 species of arthropods, in view of the obtained findings, it is conceivable that much more additional arthropod-associated *Rickettsiales* and other bacteria are still to be discovered in the studied geographic region.

Spotted fever group *Rickettsia* including *R. monacensis, R. helvetica, R. massiliae* and *R. aeschlimannii*, are known to cause spotted fever in Europe^22^. In the present study, majority of the tested pools of *I. ricinus* 62.5% (5/8) were found positive for SFG *Rickettsia* bacteria, including *R. monacensis*, *R. helvetica* and newly found *Rickettsia* sp. Up to now, there is only one previous study describing the presence of *R. monacensis* and *R. helvetica* in ixodid ticks in Serbia^23^. However, serological evidence of human rickettsial exposure in Serbia had been reported, with the prevalence of seropositivity of up to 23% in some regions^24^, in spite of no cases of clinically manifested human infection reported in Serbia so far. Together with our results, these findings imply potential for significant public health risk associated with human tick-borne rickettsial diseases, with further studies needed to estimate the conceivable risk and burden of rickettsial disease in Serbia.

Additionally, we also identified a *Rickettsia* strain from *P. complanatus*. Regardless of the known considerable prevalence of endosymbionts including *Rickettsiales* in terrestrial arthropods^4^, this is the first report on infection of arthropods of the subphylum *Myriapoda* by the *Rickettsiales* bacteria. Remarkably, this strain was distinct from those of any known *Rickettsia* in the *rrs*, *gltA* and *groEL* gene trees, with 99.62%, 98.11% and 80.52% nucleotide identity, suggesting that it may represent a novel species. Hence, these data may extend our knowledge on bacterial endosymbionts in the previously ignored Myriapoda hosts.

Further on, one of the *I. ricinus* ticks pools tested positive for *Ehrlichia* sp. HF. This bacterium has so far been known to be present mostly in Japan^25^. This is the first report of this bacterium in Serbia and among the first ones in Europe, besides France and Romania, where it was only recently described^26,27^.

More importantly, the obtained results indicate that we may have identified, through genetic detection, a Candidatus novel *Anaplasmataceae* genus in centipede *Strigamia sp*. Phylogenetic analyses of the recovered *rrs*, *groEL*, *gltA* and *rpoB* genes provided evidence for the uniqueness of the Bel-57 strain, and suggest its placement into a novel genus within the order *Rickettsiales* and the family *Anaplasmataceae*. According to the nucleotide identity and phylogenetic analysis, the *Candidatus* Neowolbachia is mostly related to the genus *Wolbachia*, which interact with host cells and induce reproductive manipulations, such as feminization, parthenogenesis, male killing, etc. However, all efforts to obtain the sequences of other key genes (*wsp*, *ftsZ*, *fbpA*, etc) in the *Candidatus* Neowolbachia serbia Bel-57 sample have failed. Notably, 16S rRNA gene sequences alone do not describe a species, but may provide the first indication that a novel species has been detected (less than 97% gene sequence similarity)^28^. A multigenic approach, as in our study, is used to improve phylogenetic and species resolution. However, the proposed assignment to a new genus is still to be considered as provisional. Noteworthy question remains whether the newly identified *Candidatus* Neowolbachia does possess homologous key genes (*wsp*, *ftsZ*, *fbpA*, etc) in the genome and whether those have similar manipulation effect on host cells as in other *Wolbachia*.

*Wolbachia* is a highly diverse genus that has been divided into 16 supergroups (A to F, H to Q) on the basis of nucleotide sequences^29^. As intracellular parasites, they maneuver the reproduction of their arthropod hosts in numerous ways, induce evolutionary alterations and are being considered as potential biological pest control. It is estimated that 66% of all insect species are infected with *Wolbachia*^5^. *W. pipientis* is a well-known endosymbiont harbored by *C. pipiens*, itself being the most ubiquitous mosquito in temperate zones. However, the presence and prevalence of mosquito infestation by *Wolbachia* has not been studied so far in the region of Serbia and this is the first study reporting about it. It has been widely accepted that the *Wolbachia* prevalence in *Culex pipiens* is 100%^30^, however, in this study the positive rate is quite low (5/18). It is supposed that this abnormal positive rate might be due to the inadequate primers for *W. pipientis*. In addition to the *W. pipientis* identified from mosquitoes and *w*Cl from bedbugs, a tentative novel *Wolbachia* strain was identified in the flea (Siphonaptera) *Ischnopsyllus* sp., collected from *N. noctula* bat. Bats are increasingly recognized as reservoirs of emerging pathogens^31,32^. This feature is underlined by their ubiquitous occurrence, long life-span, social behaviour (close contacts in colonies) and tendency for persistent infections^33^. Bats also serve as the final or intermediate host to a great diversity of ecto- and endoparasites. Because this animal group is isolated from other vertebrate species ecologically and behaviorally, their parasite species and communities are specific and quite distinct from those of other vertebrates and tend to be uniquely adapted to bats. Importantly, bats are frequently found flying and roosting in close proximity to human abodes, in a way that direct contact with bats is not a prerequisite for humans to contract bat-associated pathogens or to become infested with bat ectoparasites. Fleas are important vectors and reservoirs of several pathogens that cause emerging or re-emerging infectious diseases such as *Yersinia pestis*, *Rickettsia typhi*, *Rickettsia felis*, and *Bartonella henselae* and other dangerous pathogens^34^. *Wolbachia* spp. have also been previously detected within members of the Siphonaptera, including some bat fleas^35^. However, in spite of the estimated high prevalence of *Wolbachia* in arthropods species, to our knowledge, this is the first report of *Wolbachia* from *Ischnopsyllus* host. In the phylogenetic tree, the *w*Is Bel-A04 strain is closely related to the *w*Bs which represents the K supergroup. *Bryobia* is a genus of mites in the spider mite family, *Tetranychidae*, *Bryobia praetiosa* being perhaps the best known species. It is herbivorous, with occasional reports of it occurring as an ectoparasite on humans, causing an itchy skin irritation^36^. Isolated cases of infestation in animals have also been reported^37^. To be noticed, *Ischnopsyllus* sp. is a member of class Insecta while *Bryobia spec* belongs to class Arachnida. It is interesting that these two phylogenetically distant arthropods carry genetically related *Wolbachia* bacteria. Horizontal transfer of *Wolbachia* has been well described, both within the same species or among different species^38^, nevertheless, the occurrence of the interactions between these two vectors/hosts would not be easy to foresee.

A potential limitation of the presented study regards the choice and number of the tested arthropod species. One might argue that the included spectrum of species is insufficient and not random (only 9 species with 5 millipede species) to describe the overall variability of bacterial endosymbionts in arthropod hosts, however, the obtained results do undoubtedly provide an insight into novel host-parasite associations and give new information about geographical spread and occurrence of diverse *Rickettsiale* species.

In conclusion, 9 species of arthropods were sampled on the territory of Serbia, mostly in the capital city of Belgrade. From these samples, five recognized species and four prospective newly detected species, were recovered, including *Rickettsiaceae*, *Anaplasmataceae* and a novel *Candidatus* Neowolbachieae. The obtained results highlight considerable diversity of *Rickettsiales* bacteria in arthropods in this region. Furthermore, due to the high prevalence of SFG Rickettsia in ticks, regular surveillance on *Rickettsiales* infection should be strongly considered. This is also the first study to report the detection of Rickettsial infection in an arthropod of the subphylum *Myriapoda* (*P. complanatus)* and the *Ischnopsyllus* bat flea. In summary, the finding may shed a light into further understanding on the diversity of endosymbiotic bacteria in arthropods, and the co-evolution between bacterial endosymbionts and their hosts.

## Supplementary information

Supplementary Tables

## Data Availability

The dataset supporting the conclusions of this article are included within the article.
