# Supplementary material for: Insight into diversity of bacteria belonging to the order Rickettsiales in 9 arthropods species collected in Serbia
Source: Sci Rep. 2019 Dec 10;9:18680. doi: 10.1038/s41598-019-55077-y (PMC6904564; doi:10.1038/s41598-019-55077-y)
Supplement: Supplementary file 1 — Supplementary Tables [file 41598_2019_55077_MOESM1_ESM.docx]

**Insight into diversity of bacteria belonging to the order Rickettsiales in 9 arthropods species collected in Serbia**

Kun Li^1,+^, Maja Stanojević^2,+^, Gorana Stamenković^3^, Bojan Ilić^4^, Milan Paunović^5^, Miao Lu^1^, Branislav Pešić^6^, Ivana Đurić Maslovara^6^, Marina Siljic^2^, Valentina Cirkovic^2^, Yongzhen Zhang^1,7*^

^1^ Department of Zoonoses, National Institute for Communicable Disease Control and Prevention, Changping, Beijing, China.

^2^ University of Belgrade Faculty of Medicine, Institute of Microbiology and Immunology, Belgrade, Serbia.

^3^ University of Belgrade, Institute for Biological Research, Siniša Stanković, Department for Genetic Research, Belgrade, Serbia.

^4^ University of Belgrade, Faculty of Biology, Institute of Zoology, Department of Animal Development, Belgrade, Serbia.

^5^ Natural History Museum in Belgrade, Belgrade, Serbia.

^6^ Institute for Biocides and Medical ecology, Belgrade, Serbia.

^7^ Shanghai Public Health Clinical Center & Institute of Biomedical Sciences, Fudan University, Shanghai, China.

Table S1 The code, pool size, locality and collection date of the arthropods collected from Belgrade.

| **Pool No.** | **Pool code** | **Pool size** | **Species** | **Classification** | **Coordinates** | **Collection date** | **Description of trapping site*** |
| --- | --- | --- | --- | --- | --- | --- | --- |
| 1 | 1 | 20 | *Culex pipiens* | *Insecta, Culicidae* | N44.816 E20.534 | Aug-2016 | river bank |
| 2 | 2 | 20 | *Culex pipiens* | *Insecta, Culicidae* | N44.759 E20.625 | Jul-2016 | river bank |
| 3 | 3 | 20 | *Culex pipiens* | *Insecta, Culicidae* | N44.827 E20.467 | Aug-2016 | river bank |
| 4 | 4 | 20 | *Culex pipiens* | *Insecta, Culicidae* | N44.863 E20.370 | Jul-2016 | park |
| 5 | 5 | 20 | *Culex pipiens* | *Insecta, Culicidae* | N44.798 E20.470 | Jul-2016 | park |
| 6 | 6 | 20 | *Culex pipiens* | *Insecta, Culicidae* | N44.810 E20.244 | Aug-2016 | park |
| 7 | 7 | 20 | *Culex pipiens* | *Insecta, Culicidae* | N44.816 E20.534 | Jul-2016 | river bank |
| 8 | 8 | 20 | *Culex pipiens* | *Insecta, Culicidae* | N44.812 E20.490 | Aug-2016 | forest |
| 9 | 9 | 20 | *Culex pipiens* | *Insecta, Culicidae* | N44.803 E20.513 | Aug-2016 | park |
| 10 | 10 | 20 | *Culex pipiens* | *Insecta, Culicidae* | N44.868 E20.594 | Jul-2016 | park |
| 11 | 11 | 20 | *Culex pipiens* | *Insecta, Culicidae* | N44.766 E20.417 | Jul-2016 | park |
| 12 | 12 | 20 | *Culex pipiens* | *Insecta, Culicidae* | N44.852 E20.450 | Jul-2016 | river bank |
| 13 | 13 | 20 | *Culex pipiens* | *Insecta, Culicidae* | N44.837 E20.501 | Jul-2016 | river bank |
| 14 | 14 | 20 | *Culex pipiens* | *Insecta, Culicidae* | N44.829 E20.383 | Jul-2016 | park |
| 15 | 16 | 20 | *Culex pipiens* | *Insecta, Culicidae* | N44.869 E20.450 | Jul-2016 | river bank |
| 16 | 17 | 20 | *Culex pipiens* | *Insecta, Culicidae* | N44.881 E20.481 | Jul-2016 | river bank |
| 17 | 18 | 20 | *Culex pipiens* | *Insecta, Culicidae* | N44.869 E20.476 | Jul-2016 | river bank |
| 18 | 19 | 20 | *Culex pipiens* | *Insecta, Culicidae* | N44.810 E20.542 | Jul-2016 | park |
| 19 | 21-23 | 10 | *Ixodes ricinus* | *Arachnida, Ixodidae* | N44.766 E20.418 | Sep-2016 | park |
| 20 | 24-28 | 10 | *Ixodes ricinus* | *Arachnida, Ixodidae* | N44.778 E20.404 | Sep-2016 | forest |
| 21 | 29-32 | 10 | *Ixodes ricinus* | *Arachnida, Ixodidae* | N44.764 E20.431 | Sep-2016 | forest |
| 22 | 4000 | 1 | *Ixodes ricinus* | *Arachnida, Ixodidae* | N45.455 E19.220 | Sep-2016 | meadow |
| 23 | 4077 | 1 | *Ixodes ricinus* | *Arachnida, Ixodidae* | N42.924 E22.169 | May-2016 | mountain forest |
| 24 | 4109 | 2 | *Ixodes ricinus* | *Arachnida, Ixodidae* | N42.710 E22.342 | Jun-2016 | mountain forest |
| 25 | 4113 | 1 | *Ixodes ricinus* | *Arachnida, Ixodidae* | N42.710 E22.342 | Jun-2016 | mountain forest |
| 26 | 4100-4109 | 2 | *Ixodes ricinus* | *Arachnida, Ixodidae* | N42.710 E22.342 | Jun-2016 | mountain forest |
| 27 | 33-39- 43 | 25 | *Cimex lectularius* | *Insecta, Cimicidae* | N44.935 E21.136 | Sep-2016 | [grassland](https://www.thesaurus.com/browse/grassland) |
| 28 | 40 | 3 | *Cimex lectularius* | *Insecta, Cimicidae* | N44.935 E21.136 | Sep-2016 | [grassland](https://www.thesaurus.com/browse/grassland) |
| 29 | 41 | 3 | *Cimex lectularius* | *Insecta, Cimicidae* | N44.935 E21.136 | Sep-2016 | [grassland](https://www.thesaurus.com/browse/grassland) |
| 30 | 42 | 3 | *Cimex lectularius* | *Insecta, Cimicidae* | N44.935 E21.136 | Sep-2016 | [grassland](https://www.thesaurus.com/browse/grassland) |
| 31 | 44-52 | 31 | *Cimex lectularius* | *Insecta, Cimicidae* | N44.935 E21.136 | Sep-2016 | [grassland](https://www.thesaurus.com/browse/grassland) |
| 32 | 53 | 8 | *Polydesmus complanatus* | *Myriapoda, Polydesmidae* | N44.852 E20.450 | Jun-2016 | river bank |
| 33 | 54 | 4 | *Clinopodes flavidus* | *Myriapoda, Geophilidae* | N44.690 E20.514 | May-2016 | mountain forest |
| 34 | 55 | 9 | Cryptops anomalans | *Myriapoda, Cryptopidae* | N44.852 E20.450 | Jun-2016 | river bank |
| 35 | 56 | 7 | Cryptops anomalans | *Myriapoda, Cryptopidae* | N44.852 E20.450 | Jun-2016 | river bank |
| 36 | 57 | 4 | Strigamia bothriopus | *Myriapoda,* Linotaeniidae | N44.852 E20.450 | Jun-2016 | river bank |
| 37 | 58 | 2 | Pachyiulus hungaricus | *Myriapoda,* Julidae | N44.690 E20.514 | Jun-2016 | mountain forest |
| 38 | 59 | 10 | Cryptops anomalans | *Myriapoda,* Cryptopidae | N44.852 E20.450 | Jun-2016 | river bank |
| 39 | A04 | 9 | *Ischnopsyllus* sp. | *Insecta, Ischnopsyllidae* | N44.783 E20.507 | Ser-2016 | urban area |

Table S2 The primers used for amplification of *gltA*, *groEL* and *rpoB* genes from *Wolbachia* and *Neowolbachia* by nested PCR.

| Primer | Cycle | Bacteria | Gene | Sequence | Amplicon size |
| --- | --- | --- | --- | --- | --- |
| WoGltex5 | 1 | *Wolbachia* | *gltA* | 5-TTACATTYATYGATGGRRATGAAGG-3 | 985 bp |
| WoGltex3 | 1 | *Wolbachia* | *gltA* | 5-GGTTCTTGCAAGWGCAAARAT-3 | 985 bp |
| WoGltin5 | 2 | *Wolbachia* | *gltA* | 5-ACWKCWGTKATTYATYTATTRCTCTAT-3 | 760 bp |
| WoGltin3 | 2 | *Wolbachia* | *gltA* | 5-MYCVATAGCAKTYATTATTATACCTG-3 | 760 bp |
| WoGroex5 | 1 | *Wolbachia* | *groEL* | 5-GCARTRRTDGAYTCAMCRGT-3 | 872 bp |
| WoGroex3 | 1 | *Wolbachia* | *groEL* | 5-ATTCCAAGYTCATCYTTTATKAC-3 | 872 bp |
| WoGroin5 | 2 | *Wolbachia* | *groEL* | 5-CTAGRGGRAAWACRRTRGGRATT-3 | 614 bp |
| WoGroin3 | 2 | *Wolbachia* | *groEL* | 5-AAARCCWGRRGCTTTYACYGC-3 | 614 bp |
| RpoAex5 | 1 | *Neowolbachia* | *rpoB* | 5-GNKCWCCWGGNGTVTTYTT-3 | 1891 bp |
| RpoAex3 | 1 | *Neowolbachia* | *rpoB* | 5-GRWCCATCWGCWAKHAYATC-3 | 1891 bp |
| RpoAin5 | 2 | *Neowolbachia* | *rpoB* | 5-TYATWCCWTAYAGRGGWTCDTGG-3 | 1236 bp |
| RpoAin3 | 2 | *Neowolbachia* | *rpoB* | 5-TYTCWATRAAVCCATRYTTRTTDAT-3 | 1236 bp |
| RpoBex5 | 1 | *Neowolbachia* | *rpoB* | 5-GCTTTSYCAATTYATGGATCARAC-3 | 1805 bp |
| RpoBex3 | 1 | *Neowolbachia* | *rpoB* | 5-ARHATYTGHCCHAYATTCAT-3 | 1805 bp |
| RpoBin5 | 2 | *Neowolbachia* | *rpoB* | 5-GTGCAYCCAACKCACTAYGG-3 | 1619 bp |
| RpoBin3 | 2 | *Neowolbachia* | *rpoB* | 5-CCRTCHKCYAARWAHGGMATATC-3 | 1619 bp |
| groElex5 | 1 | *Neowolbachia* | *groEL* | 5-GARRTHACWAARGAYGGHTAYAARGT-3 | 707 bp |
| groELex3 | 1 | *Neowolbachia* | *groEL* | 5-CCRAADCCWGGWGCYTTNACWGC-3 | 707 bp |
| groELin5 | 2 | *Neowolbachia* | *groEL* | 5-AAYGAYAARGYBGGDGATGGDAC-3 | 603 bp |
| groELin3 | 2 | *Neowolbachia* | *groEL* | 5-CCYTCDAYRTCYTCHGCRATDAT-3 | 603 bp |
| Gltex5 | 1 | *Neowolbachia* | *gltA* | 5-TTACATTYATYGATGGRRATGAAGG-3 | 976 bp |
| Gltex3 | 1 | *Neowolbachia* | *gltA* | 5-ACWKCWGTKATTYATYTATTRCTCTAT-3 | 976 bp |
| Gltin5 | 2 | *Neowolbachia* | *gltA* | 5-GCAAARATDGGYGTRAACATWYTTG-3 | 738 bp |
| Gltin3 | 2 | *Neowolbachia* | *gltA* | 5-MYCVATAGCAKTYATTATTATACCTG-3 | 738 bp |

Table S3 Nucleotide identity and coverage of *Candidatus* Neowolbachia serbia Bel-57 against *Wolbachia*, *Ehrlichia*, *Rickettsia*, *Anaplasma* and *Neoehrlichia*.

|  | ***Wolbachia*** | ***Ehrlichia*** | ***Rickettsia*** | ***Anaplasma*** | ***Neoehrlichia*** |
| --- | --- | --- | --- | --- | --- |
| ***rrs*** | 91.0%/99.8% | 91.2%/99.8% | 85.5%/99.8% | 89.5%/99.8% | 90.6%/99.8% |
| ***gltA*** | 71.6%/98.8% | 67.9%/97.9% | 71.4%/59.9% | 66.7%/61.8% | 67.7%/51.6% |
| ***groEL*** | 71.3%/98.0% | 70.6%/97.0% | 70.2%/48.3% | 77.1%/62.3% | 71.8%/83.3% |
| ***rpoB*** | 74.1%/92.1% | 73.9%/100% | 69.4%/62.9% | 70.6%/90.8% | 76.6%/5.1% |

Table S4 Genbank numbers of bacterial sequences used in phylogenetic analysis.

| Gene | Bacterial strain | Genbank number |
| --- | --- | --- |
| 16S | Rickettsia africae str. ESF-5 | L36098 |
| 16S | Rickettsia akari str. Hartford | U12458 |
| 16S | Rickettsia amblyommii str. MO 85-1084 | U11012 |
| 16S | Rickettsia asiatica str. IO-1 | NR041840 |
| 16S | Rickettsia australis str. Phillips | U12459 |
| 16S | Rickettsia bellii str. 369-C | U11014 |
| 16S | Rickettsia buchneri str. ISO7 | NR134842 |
| 16S | Rickettsia canadensis str. 2678 | NR029155 |
| 16S | Rickettsia conorii | L36107 |
| 16S | Rickettsia endosymbiont of Deronectes platynotus | FM177877 |
| 16S | Rickettsia endosymbiont of Ixodes pacificus isolate CP-1 | KP276591 |
| 16S | Rickettsia endosymbiont of Ixodes scapularis | CM000770 |
| 16S | Rickettsia endosymbiont of Pnigalio sp. | EU881508 |
| 16S | Rickettsia helvetica str. C9P9 | L36212 |
| 16S | Rickettsia felis str. scc50 | DQ102712 |
| 16S | Rickettsia heilongjiangensis str. 054 | NR074469 |
| 16S | Rickettsia japonica str. YH | NR074459 |
| 16S | Rickettsia limoniae str. Gent | AF322442 |
| 16S | Rickettsia marmionii str. KB | AY737685 |
| 16S | Rickettsia massiliae str. Mtu1 | NR025919 |
| 16S | Rickettsia monacensis str. IrR/Munich | NR115686 |
| 16S | Rickettsia montana str. ATCC VR-611 | L36215 |
| 16S | Rickettsia parkeri str. Portsmouth | CP003341 |
| 16S | Rickettsia peacockii str. Rustic | DQ062433 |
| 16S | Rickettsia prowazekii str. NMRC Madrid E | CP004888 |
| 16S | Rickettsia raoultii isolate BL029-2 | KJ410261 |
| 16S | Rickettsia rickettsii str. Iowa | CP018914 |
| 16S | Rickettsia sibirica str. ATCC VR151 | D38628 |
| 16S | Rickettsia slovaca str. 13-B | NC016639 |
| 16S | Rickettsia sp. str. IRS4 | AF141908 |
| 16S | Rickettsia symbiont of Nephotettix cincticeps | AB702995 |
| 16S | Rickettsia tamurae strain AT-1 | NR042727 |
| 16S | Rickettsia typhi str. Wilmington | NR118679 |
| 16S | Anaplasma centrale | AF283007 |
| 16S | Anaplasma marginale | M60313 |
| 16S | Anaplasma ovis str. Idaho | AF309865 |
| 16S | Anaplasma phagocytophilum str. Webster | NR044762 |
| 16S | Ehrlichia sp. HF | DQ647318 |
| 16S | Ehrlichia chaffeensis str. Arkansas | NR074500 |
| 16S | Ehrlichia muris str. AS145 | NR025962 |
| 16S | Neorickettsia helminthoeca | U12457 |
| 16S | Neorickettsia risticii str. Illinoi | NR029162 |
| 16S | Wolbachia endosymbiont of Cimex lectularius | AP013028 |
| 16S | Wolbachia endosymbiont of Culex quinquefasciatus Pel strain wPip | AM999887 |
| 16S | Wolbachia endosymbiont of Drosophila simulans strain wRi | DQ412085 |
| 16S | Wolbachia endosymbiont of Bryobia spec. I VIDR-2008 str. NL14 | EU499318 |
| 16S | Wolbachia endosymbiont of Mansonella ozzardi | AJ279034 |
| 16S | Wolbachia endosymbiont of Mesaphorura yosii isolate wMyos | KT799588 |
| 16S | Wolbachia endosymbiont of Myodopsylla gentilis | AY335918 |
| 16S | Wolbachia endosymbiont of Onchocerca volvulus str. Cameroon | HG810405 |
| 16S | Wolbachia endosymbiont of Pentalonia nigronervosa | KJ786952 |
| 16S | Gut symbionts of *Harpalus pensylvanicus* clone F1 | GU815101 |
| 16S | Gut symbionts of *Harpalus pensylvanicus* clone S(a)3 | GU815124 |
| gltA | Rickettsia asiatica str. IO-1 | AF394901 |
| gltA | Rickettsia bellii | DQ146481 |
| gltA | Rickettsia endosymbiont of Ixodes pacificus isolate CP-1 | KP276598 |
| gltA | Rickettsia endosymbiont of Ixodes scapularis | KY678090 |
| gltA | Rickettsia endosymbiont Quadrastichus mendeli | KX673387 |
| gltA | Rickettsia felis | JQ674484 |
| gltA | Rickettsia heilongjiangensis | AY285776 |
| gltA | Rickettsia helvetica | KU310588 |
| gltA | Rickettsia japonica str. Inha1 | AY743327 |
| gltA | Rickettsia monacensis strain IrR/Munich | DQ100163 |
| gltA | Rickettsia parkeri str. Portsmouth | CP003341 |
| gltA | Rickettsia peacockii str. Rustic | DQ100162 |
| gltA | Rickettsia raoultii isolate BL029-2 | KJ410264 |
| gltA | Rickettsia rickettsii str. Iowa | CP018914 |
| gltA | Rickettsia sibirica | HM050279 |
| gltA | Rickettsia slovaca str. 13-B | U59725 |
| gltA | Rickettsia sp. IRS 4 | AF141906 |
| gltA | Rickettsia symbiont of Nephotettix cincticeps | KU586334 |
| gltA | Anaplasma bovis | MH594293 |
| gltA | Anaplasma centrale | AF304141 |
| gltA | Anaplasma marginale | KU586328 |
| gltA | Anaplasma ovis | KX579068. |
| gltA | Anaplasma phagocytophilum | KP861639 |
| gltA | Ehrlichia chaffeensis str. Arkansas | AF304142 |
| gltA | Ehrlichia ewingii | DQ365879 |
| gltA | Ehrlichia sp. HF | DQ647319 |
| gltA | Wolbachia endosymbiont of Acraea eponina | DQ266532 |
| gltA | Wolbachia endosymbiont of Bryobia spec. I VIDR-2008 strain NL14 | EU499328 |
| gltA | Wolbachia endosymbiont of Caudra cautella | DQ266530 |
| gltA | Wolbachia endosymbiont of Cimex lectularius | AP013028 |
| gltA | Wolbachia endosymbiont of Culex quinquefasciatus Pel strain wPip | AM999887 |
| gltA | Wolbachia endosymbiont of Drosophila simulans wHa | CP003884 |
| gltA | Wolbachia endosymbiont of Onchocerca volvulus str. Cameroon | HG810405 |
| gltA | Wolbachia endosymbiont of Protocalliphora sialia str. A1 | DQ266412 |
| groEL | Rickettsia bellii str. RML369-C | CP000087 |
| groEL | Rickettsia bellii isolate An04 | CP015010 |
| groEL | Rickettsia endosymbiont of Bemisia tabaci | EU435143 |
| groEL | Rickettsia endosymbiont of Ixodes pacificus strain Humboldt reip | LAOP01000001 |
| groEL | Rickettsia endosymbiont of Ixodes scapularis | CM000770 |
| groEL | Rickettsia felis str. LSU-Lb | JSEL01000012 |
| groEL | Rickettsia heilongjiangensis str. 054 | CP002912 |
| groEL | Rickettsia helvetica | DQ442911 |
| groEL | Rickettsia japonica str. LA16/2015 | KY073365 |
| groEL | Rickettsia monacensis str. IrR/Munich | LN794217 |
| groEL | Rickettsia parkeri str. Portsmouth | CP003341 |
| groEL | Rickettsia peacockii str. Rustic | CP001227 |
| groEL | Rickettsia prowazekii str. Dachau | CP003394 |
| groEL | Rickettsia rickettsii str. Iowa | CP018914 |
| groEL | [Rickettsia slovaca str. 13-B](https://www.ncbi.nlm.nih.gov/nuccore/NC_016639.1) | NC016639 |
| groEL | Anaplasma bovis | MH255898 |
| groEL | Anaplasma centrale | KY523001 |
| groEL | Anaplasma ovis | AF441131 |
| groEL | Anaplasma phagocytophilum | KF015601 |
| groEL | Anaplasma platys | MH716435 |
| groEL | Anaplasma sp. clone SY49 | KF728361 |
| groEL | Ehrlichia chaffeensis | JQ085941 |
| groEL | Ehrlichia ewingii | AF195273 |
| groEL | Ehrlichia sp. HF | CP007474 |
| groEL | Candidatus Neoehrlichia australis | KU865440 |
| groEL | Candidatus Neoehrlichia lotoris str. RAC413 | EF633745 |
| groEL | Candidatus Neoehrlichia mikurensis | EU810406 |
| groEL | Wolbachia endosymbiont of Cercopithifilaria japonica | FR827917 |
| groEL | Wolbachia endosymbiont of Cimex lectularius | AP013028 |
| groEL | Wolbachia endosymbiont of Culex quinquefasciatus Pel strain wPip | AM999887 |
| groEL | Wolbachia endosymbiont of Drosophila simulans str. wHa | CP003884 |
| groEL | Wolbachia endosymbiont of Litomosides sigmodontis | AF409113 |
| groEL | Wolbachia endosymbiont of Nasutitermes sp. isolate 86YT MNHN | KU255222 |
| groEL | Wolbachia endosymbiont of Onchocerca volvulus str. Cameroon | HG810405 |
| rpoB | Rickettsia bellii str. RML369-C | CP000087 |
| rpoB | Rickettsia conorii str. Moroccan | AF076435 |
| rpoB | Rickettsia monacensis str. IrR/Munich | LN794217 |
| rpoB | Rickettsia prowazekii | AF034531 |
| rpoB | Rickettsia pulicis | AF236795 |
| rpoB | Rickettsia slovaca 13-B | NC016639 |
| rpoB | Rickettsia typhi | AF083622 |
| rpoB | Anaplasma marginale | AF389472 |
| rpoB | Anaplasma ovis str. Haibei | CP015994 |
| rpoB | Anaplasma phagocytophilum | AF237414 |
| rpoB | Ehrlichia canis strain YZ-1 | CP025749 |
| rpoB | Ehrlichia chaffeensis | AF389473 |
| rpoB | Ehrlichia minasensis str. B11 | QOHL01000015 |
| rpoB | Ehrlichia muris str. AS145 | CP006917 |
| rpoB | Ehrlichia ruminantium str.Welgevonden | CR767821 |
| rpoB | Neorickettsia risticii | AF401089 |
| rpoB | Neorickettsia sennetsu | AF401088 |
| rpoB | Wolbachia endosymbiont of Onchocerca ochengi | NC018267 |
| rpoB | Wolbachia pipientis | AF401090 |

Table S5 Nucleotide identity of the *Rickettsiale* sequences obtained in this study against reference sequences in Genbank.

|  | ***Rickettsia monacensis*** | ***Rickettsia helvetica*** | ***Rickettsia* spp.** | ***Rickettsia* endosymbiont**  **of *Polydesmus complanatus*** | ***Ehrlichia sp. HF*** | ***Wolbachia pipientis*** | ***Wolbachia* endosymbiont of *Cimex lectularius*** | ***Wolbachia* endosymbiont of *Ischnopsyllus* sp.** | ***Candidatus***  **Neowolbachia serbia** |
| --- | --- | --- | --- | --- | --- | --- | --- | --- | --- |
| ***rrs*** | 100%  *Rickettsia monacensis* | 100%  *Rickettsia helvetica* | 99.85%  *Rickettsia raoultii* | 99.62%  *Rickettsia* symbiont of *Nephotettix cincticeps* | 100%  *Ehrlichia sp. HF* | 100%  *Wolbachia pipientis* | 99.92%  *Wolbachia* endosymbiont of *Cimex lectularius* | 98.31%  *Wolbachia* endosymbiont of *Bryobia spec* | 93.84%  Gut symbionts of *Harpalus pensylvanicus* |
| ***gltA*** | 99.3%  *Rickettsia sp.* | 100%  *Rickettsia helvetica* | 99.65%  [*Rickettsia parkeri*](https://blast.ncbi.nlm.nih.gov/Blast.cgi#alnHdr_1655365547) | 98.11%  *Rickettsia* symbiont of *Nephotettix cincticeps* | 99.8%  *Ehrlichia sp. HF* | 100%  *Wolbachia endosymbiont of Culex quinquefasciatus* | 100%  *Wolbachia* endosymbiont of *Cimex lectularius* | 93.82%  *Wolbachia* endosymbiont of *Bryobia spec* | 71.60%  *Wolbachia* endosymbiont of *Culex quinquefasciatus* |
| ***groEL*** | 100%  *Rickettsia monacensis* | 99%  *Rickettsia asiatica* | 99.63%  *Rickettsia rickettsii* | 80.52%  *Rickettsia bellii* | 99.51%  *Ehrlichia sp. HF* | 99.51%  *Wolbachia endosymbiont of Culex quinquefasciatus* | 99.51%  *Wolbachia* endosymbiont of *Cimex lectularius* | 87.56%  *Wolbachia* endosymbiont of *Nasutitermes sp.* | 71.31%  *Wolbachia* endosymbiont of *Folsomia candida* |
